# Screening and quantification of anti-quorum sensing and antibiofilm activities of phyllosphere bacteria against biofilm forming bacteria

**DOI:** 10.1186/s13104-019-4775-1

**Published:** 2019-11-07

**Authors:** Nadine Amabel Theodora, Vania Dominika, Diana Elizabeth Waturangi

**Affiliations:** Faculty of Biotechnology, Atma Jaya Catholic University of Indonesia, Jenderal Sudirman 51 Street, South Jakarta, 12930 DKI Jakarta, Indonesia

**Keywords:** Antibiofilm activity, *Chromobacterium violaceum*, Phyllosphere, Quorum sensing, Violacein

## Abstract

**Objective:**

The objectives of this research were to screen anti-quorum sensing activity of phyllosphere bacteria and quantify their antibiofilm activity against biofilm forming bacteria (*Bacillus cereus, Staphylococcus aureus, Enterococcus faecalis, Salmonella typhimurium, Vibrio cholerae, Pseudomonas aeruginosa*).

**Results:**

We found 11 phyllosphere bacteria isolates with potential anti-quorum sensing activity. Most of the crude extracts from phyllosphere bacteria isolates had anti-quorum sensing activity against *Chromobacterium violaceum* at certain concentration (20 and 10 mg/mL), but not crude extract from isolate JB 7F. Crude extract showed the largest turbid zone (1,27 cm) using isolate JB 14B with concentration of 10 mg/mL and the narrowest turbid zone isolate (1 cm) using JB 18B with concentration of 10 mg/mL. Crude extracts showed various antibiofilm activities against all tested pathogenic bacteria, it showed the highest biofilm inhibition (90%) and destruction activities (76%) against *S. aureus*.

## Introduction

Nowadays, we know that about 65% of all bacterial infections were associated with bacterial biofilms [1]. Biofilm is an organized aggregate of microorganisms like bacteria within an extracellular polymeric matrix that they produce [1, 2]. Several pathogenic bacteria form a biofilm using a mechanism called quorum sensing. Quorum sensing is a communication form among bacteria by various types of extracellular signal molecules called autoinducer (AI). Bacteria in biofilm were more resistant to antibiotic because biofilm matrix can help with interfering the penetration of antibiotic. Therefore we need to explore compound that have capability to inhibit or destroy biofilm as well as anti-quorum sensing to control attack of biofilm-forming pathogenic bacteria [3].

Phyllosphere bacteria, which lives the most on the leaves surface area, reported to have potential in quorum quenching activity with produce molecules such as AHL lactonase enzyme [4–6]. High populations of phyllosphere bacteria show activities such as antimicrobial and antibiofilm that produced to survive on the leaves surface area [7]. Many research have been conducted to analyze anti-quorum sensing activity from phyllosphere bacteria. The objectives of this research were to screen anti-quorum sensing activity of phyllosphere bacteria using *Chromobacterium violaceum* as indicator bacteria and quantify their antibiofilm activity against biofilm forming bacteria (*Bacillus cereus, Staphylococcus aureus, Enterococcus faecalis, Salmonella typhimurium, Vibrio cholerae, Pseudomonas aeruginosa*).

## Main text

### Methods

#### Bacterial cultivation

The phyllosphere bacteria were from Atma Jaya Catholic University of Indonesia culture collections in cryopreservation. These bacteria were from previous research and recovered from *Psidium guajava*, *Averrhoa carambola*, and *Anredera cordifolia* leaves [8, 9]. Bacteria were grown in Luria–Bertani Agar (LA) and were incubated at 28 °C for 48 h. After that, single colony was picked and grown in King’s B medium and incubated at 28 °C for 48 h.

Pathogenic bacteria used were *B. cereus* ATCC 14579, *S. aureus* ATCC 29213, *E. faecalis* ATCC 33186, *P. aeruginosa* ATCC 27853, *S. typhimurium*, *V. cholerae*. All pathogenic bacteria were from cryopreservation and were streaked onto LA then incubated 37 °C overnight.

#### Primary screening of anti-quorum sensing activity

The monitor strain *C. violaceum* was grown separately in 50 mL of LB broth medium and incubated at 28 °C, 120 rpm for 48 h. Phyllosphere bacteria were streaked onto LA in a straight line then incubated at 28 °C for 24 h. After that, 100 μL of monitor strain (OD_600_ = 0.132) were put into 2 mL semisolid agar (0.75% agar) for overlay on top of the phyllosphere isolates which had been streaked before. These plates were incubated at 28 °C overnight. A positive result indicated by inhibiting violacein pigmentation (opaque zone) of the *C. violaceum* around the streak of the phyllosphere isolates [10].

#### Production of crude extract

Isolates that had given positive result from the primary screening of anti-quorum sensing activity were extracted by using liquid–liquid extraction. The bacterial culture were inoculated into 100 mL of Luria–Bertani Broth (LB) then incubated in orbital shaker incubator at 28 °C for 48 h 120 rpm. After that, centrifuged at 13,888×*g* for 15 min and cell-free supernatant was harvested and mixed with an equal volume of ethyl acetate. The solvent layer was harvested and evaporated in a rotary evaporator. After that, extract evaporated in an oven vacuum overnight to obtain the crude extract. To this, 1% of Dimethyl Sulfoxide (DMSO) will be added to obtain a final concentration of 5, 10, and 20 mg/mL stock (w/v) and kept at − 20 °C [11].

#### Antibacterial activity assay

The crude extracts that had been obtained were tested against pathogenic bacteria such as *B. cereus, E. faecalis, and S. aureus, P. aeruginosa, S. typhimurium, and V. cholerae* using agar well diffusion method. Pathogenic bacteria were streaked continuously on Brain Heart Infusion Agar (BHIA). Then, the extracts were applied 50 μL of 5, 10, and 20 mg/mL solution to the well. Streptomycin (Merck; 10 mg/mL) were used as positive control, whereas DMSO was used as negative control. The plates were incubated at 37 °C for 24 h. This assay was performed in triplicate [12].

#### Detection of anti-quorum sensing activity

The crude extracts were tested for anti-quorum sensing activity against *C. violaceum* by agar well diffusion method. *C. violaceum* was streaked on LA with a sterile cotton swab. Then the extracts (50 μL) with various concentration (5, 10, and 20 mg/mL) were applied to the well. DMSO was used as a control. The plates were incubated at 28 °C for 24 h. Anti-quorum sensing activity was observed through a turbid halo zone against a background of violacein pigment. This assay was performed in triplicate [10].

#### Quantification of antibiofilm activity

The pathogenic bacteria were inoculated into BHIB and incubated overnight. After that, for biofilm inhibition test, 100 µL of crude extracts and 100 µL of bacterial cultures (OD_600_ = 0.132) were transferred into 96-well microtiter plates (polystyrene) then incubated at 37 °C for 24 h. Meanwhile for biofilm destruction test, 100 µL of bacterial culture were transferred into 96-well microtiter plates then incubated. After that, 100 µL of crude extracts will be added and incubated at 37 °C for 24 h. Then planktonic cells and media were discarded. Adherent cells were rinsed gently twice with distilled water and allowed to air dry. The biofilms were stained by 200 μL of 0.4% (w/v) crystal violet solution for 30 min. After that, the dye were discarded and the wells were rinsed twice with distilled water. The wells were air dried and then 200 µL of ethanol were used to solubilize the crystal violet. The optical density were determined at 595 nm using a microplate reader. BHIB was used as blank and bacterial cultures without extracts were used as control. This test was performed triplicate [13].

$$Percentage \;biofilm \;inhibition = \frac{{\left( {Control \;OD595 - Treated\; OD595} \right)}}{{\left( {Control\; OD595} \right) }} \times 100\%$$

#### Microscopic observations

This step was done using Scanning Electron Microscope (SEM) at Dexa Laboratories of Biomolecular Sciences. First, *B. cereus* and *S. typhimurium* were grown in BHIB and incubated overnight. Then, bacteria were spotted to a steril cover glass and incubated overnight to form biofilm. After that, crude extracts were spotted into the biofilm and incubated at 37 °C overnight. At the last step, the results were analyzed using SEM at DLBS [14].

### Results

#### Primary screening of anti-quorum sensing activity

There were 11 out of 60 phyllosphere isolates from previous study showed an anti-quorum sensing activity. Those positive isolates were extracted and continued to the next step.

#### Antibacterial activity assay

From this assay, we know that 1 out of 11 positive phyllosphere isolates crude extract, EJB 7B, showed antibacterial activity against all Gram positive pathogenic bacteria which used in this research. Meanwhile, control positive (Streptomycin) showed various turbid zone depending on the pathogen bacteria used. Average clear zone *V. cholerae* is 2 cm, *P. aeruginosa* is 3 cm, *S. typhimurium* is 2.3 cm. Average clear zone *B. cereus* is 4 cm, *S. aureus* is 3.5 cm, *E. faecalis* is 3 cm.

#### Detection of anti-quorum sensing activity

We found out that each of phyllosphere isolate has their own optimal concentrations but most of them showed activity at concentration of 20 mg/mL and all of them have no activity at concentration of 5 mg/mL Crude extract showed the largest turbid zone (1.27 cm) using isolate JB 14B with concentration of 10 mg/mL and the narrowest turbid zone isolate (1 cm) using JB 18B with concentration of 10 mg/mL (Table 1).

**Table 1:** Result of detection of anti-quorum sensing activity

Phyllosphere isolates	Origin of isolates	Concentrations (cm)		
		5 mg/mL	10 mg/mL	20 mg/mL
JB 3B	*Psidium guajava*	0	0	0.83
JB 11B	*Psidium guajava*	0	0	1.13
JB 14B	*Psidium guajava*	0	2	1.1
JB 15B	*Psidium guajava*	0	0	1.4
JB 16B	*Psidium guajava*	0	0	1.27
JB 18B	*Psidium guajava*	0	1	1.2
JB 19B	*Psidium guajava*	0	0	1.07
JB 20B	*Psidium guajava*	0	1.2	1.7
AF3	*Anredera cordifolia*	0	0	1.1
JB 7F	*Psidium guajava*	0	0	0

#### Quantification of biofilm activity

According to the result of quantification of biofilm (inhibition) activity assay, the results showed that crude extracts had various inhibition activity against all pathogenic bacteria used in this study, with the most positive results against *S. aureus* and the least against *P. aeruginosa*. (Table 2). Crude extracts that showed the highest biofilm inhibition activity against *S. aureus* (90%) is from isolate JB 19B.

**Table 2:** Results of biofilm activity quantification against pathogenic bacteria

Pathogens	Activity	Isolates activity (%)										
		JB 3B	JB 11B	JB 14B	JB 15B	JB 16B	JB 18B	JB 19B	JB 20B	AF3	JB 7F	EJB 7B
*S. aureus*	Inhibition	87	61	67	80	72	65	90	58	35	86	X
	Destruction	73	74	0	72	62	76	59	65	23	2	X
*E. faecalis*	Inhibition	19	0	42	0	0	27	0	0	0	0	X
	Destruction	56	4	54	71	35	64	45	23	37	0	X
*B. cereus*	Inhibition	67	30	0	42	58	0	0	34	0	0	X
	Destruction	45	55	0	26	10	23	9	9	0	0	X
*V. cholerae*	Inhibition	87	0	14	0	56	18	0	80	0	0	63
	Destruction	71	72	0	58	59	48	73	0	0	0	0
*S. typhimurium*	Inhibition	29	8	37	21	33	0	0	27	8	0	5
	Destruction	0	4	0	11	11	0	23	9	15	2	12
*P. aeruginosa*	Inhibition	68	0	0	0	0	0	0	0	0	0	0
	Destruction	20	4	0	0	0	0	23	0	0	0	0

X: no test was performed

Meanwhile, different results were obtained from quantification of biofilm (destruction) activity assay. According to biofilm destruction activity data (Table 2), the results showed that crude extracts had various destruction activity against all pathogenic bacteria used in this study, with the most positive results against *S. aureus* and *E. faecalis* and the least against *P. aeruginosa* (Table 2). Crude extracts showed the highest biofilm destruction activity against *S. aureus* (76%) using isolate JB 18B.

#### Microscopic observations

Regarding the results of biofilm destruction we can determined morphological changing, which destruction activity of extract from isolate JB 18B and JB 19 B against mature biofilm of *B. cereus* and *S. typhimurium*.

## Discussion

Based on primary screening of anti-quorum sensing activity results, we found 11 out of 60 phyllosphere isolates were potential to be used as anti-quorum sensing agent. It might be happened because phyllosphere bacteria need survival strategy in the stressful environment due to the fluctuations in physical conditions and limited and highly heterogenous availability of nutrients [15].

Based on antibacterial activity assay result, we found that only isolate EJB 7B extract had antibacterial activity against Gram positive-biofilm forming bacteria. The result showed that most of them had no bactericidal activity towards biofilm-forming pathogenic bacteria which is would not lead to antibiotic resistance [10].

Based on the results, at 5 mg/mL concentration all of the phyllosphere extracts have no activity. It might be due to because the concentration were relatively small. JB 7F extract showed no activity at any concentrations because it needed higher concentration for quorum quenching activity. In this study, inhibition of violacein pigments could happened because AHL from *C. violaceum* were degraded by metabolites that produce by phyllosphere bacteria [16]. We also can conclude that quorum quenching activity is affected by bacteria producer and extract concentration that we used [10].

Biofilm is a cell function whose gene expression is regulated by quorum sensing [17, 18]. Therefore, quorum quenching mechanisms might be a good alternative to overcome biofilm problems [19]. Based on quantification of biofilm activity both in inhibition and destruction steps (Table 2), these extracts showed various results. The biofilm inhibition activity might happen because quorum sensing process of pathogenic bacteria was disturbed by interfering autoinducer synthesis, cell to cell exchange, autoinducer’s reception and transduction, and degrading autoinducer [7, 20].

The biofilm destruction activity might be the result of enzyme that could hydrolyze the compound of biofilm or small molecule that induce biofilm destruction [21]. EPS composition of pathogenic bacteria biofilms were diverse depending on the bacteria [22]. Various EPS compounds can be degraded by specific enzymes like proteases, deoxyribonucleases, glycoside hydrolase [23].

**Fig. 1:**
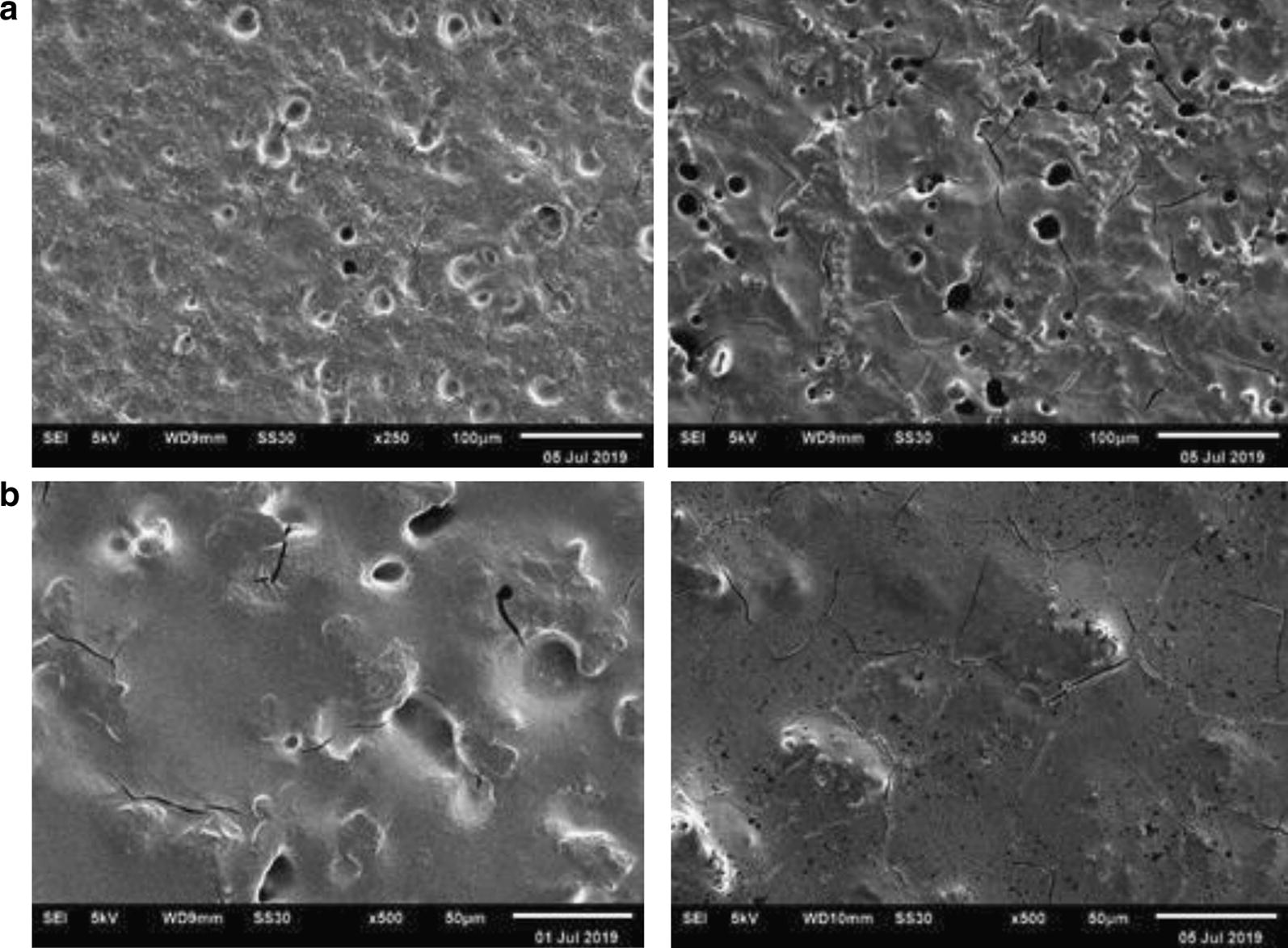
SEM images of *S. typhimurium* biofilm destruction by extract of isolate JB 19B with (**a**) pathogen control and (**b**) control + extract (×250) and *Bacillus cereus* biofilm destruction by extract of isolate JB 18B with (**a**) pathogen control and (**b**) control + extract (×500)

From SEM analysis, we can determine morphological changing which showed destruction activity (Fig. 1). It indicated there is reduction of extracellular matrix and this result approved quantification of antibiofilm assay [14]. Therefore, phyllosphere bacteria extract such as JB 18B and JB 19B can destruct biofilm of pathogenic bacteria like *B. cereus* and *S. typhimurium*.

## Conclusion

JB 3B isolate has a broad spectrum antibiofilm activity both in inhibition and destruction ways because it can inhibit and destruct almost all biofilm of pathogenic bacteria that are used in this study. So far crude extracts of phyllosphere isolates are potential to be used as quorum quenching and antibiofilm agents against some of biofilm-forming pathogenic bacteria used in this study. For future research it might be possible to sequence the phyllosphere bacteria metabolites so we can know what kind of quorum quenching agents that phyllosphere bacteria has.

## Limitation

This research did not know kind of molecule in the phyllosphere bacteria extract and did not look for at least genus of the phyllosphere bacteria.

## Data Availability

The data of this study is available with the corresponding author up on request.

## Abbreviations

AI: autoinducer
SEM: scanning electron microscope

## References

[1] Jamal M, Ahmad W, Andleeb S, Jalil F, Imran M, Nawaz MA, Hussain T, Ali M, Rafiq M, Kamil MA (2018). Bacterial biofilm and associated infections. JCMA..

[2] Hall CW, Mah T (2017). Molecular mechanisms of biofilm-based antibiotic resistance and tolerance in pathogenic bacteria. FEMS Microbiol Rev..

[3] Basavaruju M, Sisnity VS, Palaparthy R, Addanki PK (2016). Quorum quenching: signal jamming in dental plaque biofilms. J Dent Sci..

[4] Ma A, Lv D, Zhuang X, Zhuang G (2013). Quorum quenching in culturable phyllosphere bacteria from tobacco. Int J Mol Sci..

[5] Remus-Emsermann MNP, Schlechter RO (2018). Phyllosphere microbiology: at the interface between microbial individuals and the plant host. New Phytol..

[6] Satwika TD, Rusmana I, Akhdiya A (2017). Potensi quorum quenching bakteri filosfer dan rizosfer terhadap dickeya dadantii. J AgroBiogen..

[7] Zhou J, Bi S, Chen H, Chen T, Yang R, Li M, Fu Y, Jia A (2017). Anti-biofilm and antivirulence activities of metabolites from *Plectospaerella cucumerina* against *Pseudomonas aeruginosa*. Front Microbiol..

[8] Juliana. (2011). Screening of phyllosphere and endophytic microbes producing antibacterial or anti quorum sensing activity from *Ageratum conyzoides, Coleus amboinicus*, and *Psidium guajava* [skripsi].

[9] Listiarini H (2011). Screening for endophytic and phyllosphere microbes with antibacterial activity from *Centella asiatica, Mirabilis jalapa*, and *Averrhoa bilimbi* [skripsi].

[10] Abudoleh SM, Mahasneh AM (2017). Anti-quorum sensing activity of substances isolated from wild berry associated bacteria. Avicenna J Med Biotech..

[11] Younis KM, Usup G, Ahmad A (2015). Secondary metabolites produced by marine streptomyces as antibiofilm and anti-quorum sensing inhibitor of uropathogen *Proteus mirabilis*. Environ Sci Pollut Res..

[12] Tabbouche SA, Gurgen A, Yildiz S, Kilic AO, Sokmen M (2017). Antimicrobial and antiquorum sensing activity of some wild mushrooms collected from Turkey. MSU J Sci..

[13] Lokegaonkar SP, Nabar BM (2017). In vitro antibiofilm, antiquorum sensing activity of gamma tolerant streptomyces against Gram negative pathogens. Int J Pharm Clin Res..

[14] Luo J, Dong B, Wang K, Cai S, Liu T, Cheng X, Lei D, Chen Y, Kong J, Chen Y (2017). Baicalin inhibits biofilm formation, attenuates the quorum sensing-controlled virulence and enhances *Pseudomonas aeruginosa* clearance in a mouse peritoneal implant infection model. PLoS ONE..

[15] Hunter PJ, Hand P, Pink D, Whipps JM, Bending GD (2010). Both leaf properties and microbe–microbe interactions influence within-species variation in bacterial population diversity and structure in the lettuce (*Lactuca* species) phyllosphere. Appl Environ Microbiol..

[16] Stauff DL, Bassler BL (2011). Quorum sensing in Chromobacterium violaceum: DNA recognition and gene regulation by the CviR receptor. ASM..

[17] Biradar B, Devi P (2011). Quorum sensing in plaque biofilms: challenges and future prospects. JCDP..

[18] Roy R, Tiwari M, Donelli G, Tiwari V (2018). Strategies for combating bacterial biofilms: a focus on anti-biofilm agents and their mechanisms of action. Virulence..

[19] Shah S, Gaikwad S, Nagar S, Kulshretha S, Vaidya V, Nawani N, Pawar S (2019). Biofilm inhibition and anti-quorum sensing activity of phytosynthesized silver nanoparticles against the nosocomial pathogen *Pseudomonas aeruginosa*. Biofouling..

[20] Grandclement C, Tannieres M, Morera S, Dessaux Y, Faure D (2016). Quorum quenching: role in nature and applied developments. FEMS microbiol Rev..

[21] You JL, Xue XL, Cao LX, Lu X, Wang J, Zhang LX, Zhou SN (2007). Inhibition of Vibrio biofilm formation by a marine actinomycete strain A66. Appl Microbiol Biotechnol..

[22] Gunn JS, Bakaletz LO, Wozniak DJ (2016). What’s on the outside matters: the role of the extracellular polymeric substance of Gram-negative biofilms in evading host immunity and as a target for therapeutic intervention. J Biol Chem..

[23] Fleming D, Rumbaugh KP (2017). Approaches to dispersing medical biofilms. Microorganisms..

